# Supplementary material for: Iterative improvement in the automatic modular design of robot swarms
Source: PeerJ Comput Sci. 2020 Dec 7;6:e322. doi: 10.7717/peerj-cs.322 (PMC7924708; doi:10.7717/peerj-cs.322)
Supplement: Supplemental Information 3 [file peerj-cs-06-322-s003.zip › argos3/doc/api/standalone/a00618_source.html]

ARGoS: plugins/simulator/physics\_engines/dynamics2d/dynamics2d\_velocity\_control.h Source File


- Main Page
- Related Pages
- Namespaces
- Classes
- Files

- File List
- File Members

# plugins/simulator/physics\_engines/dynamics2d/dynamics2d\_velocity\_control.h

Go to the documentation of this file.

```
00001 
00007 #ifndef DYNAMICS2D_VELOCITY_CONTROL_H
00008 #define DYNAMICS2D_VELOCITY_CONTROL_H
00009 
00010 namespace argos {
00011    class CDynamics2DEngine;
00012 }
00013 
00014 #include <argos3/plugins/simulator/physics_engines/dynamics2d/chipmunk-physics/include/chipmunk.h>
00015 #include <argos3/core/utility/math/vector2.h>
00016 
00017 namespace argos {
00018 
00019    class CDynamics2DVelocityControl {
00020 
00021    public:
00022       
00023       CDynamics2DVelocityControl(CDynamics2DEngine& c_engine,
00024                              Real f_max_force,
00025                              Real f_max_torque);
00026 
00027       virtual ~CDynamics2DVelocityControl();
00028 
00029       void AttachTo(cpBody* pt_body);
00030 
00031       void Detach();
00032 
00033       void Reset();
00034 
00035       CVector2 GetLinearVelocity() const;
00036 
00037       void SetLinearVelocity(const CVector2& c_velocity);
00038 
00039       Real GetAngularVelocity() const;
00040 
00041       void SetAngularVelocity(Real f_velocity);
00042 
00043       inline Real GetMaxForce() const {
00044          return m_fMaxForce;
00045       }
00046 
00047       inline Real GetMaxTorque() const {
00048          return m_fMaxTorque;
00049       }
00050 
00051    protected:
00052 
00053       CDynamics2DEngine& m_cDyn2DEngine;
00054 
00055       cpBody* m_ptControlBody;
00056 
00057       cpBody* m_ptControlledBody;
00058 
00059       cpConstraint* m_ptLinearConstraint;
00060 
00061       cpConstraint* m_ptAngularConstraint;
00062 
00063       Real m_fMaxForce;
00064 
00065       Real m_fMaxTorque;
00066    };
00067 
00068 }
00069 
00070 #endif
```

---

Generated on 10 Jul 2018 for ARGoS by 
 1.6.1
